# Vectorial status and insecticide resistance of *Anopheles funestus *from a sugar estate in southern Mozambique

**DOI:** 10.1186/1756-3305-4-16

**Published:** 2011-02-09

**Authors:** R Graham Kloke, Eduardo Nhamahanga, Richard H Hunt, Maureen Coetzee

**Affiliations:** 1 Malaria Entomology Research Unit, School of Pathology, Faculty of Health Sciences, University of the Witwatersrand, Johannesburg, South Africa; 2 Vector Control Reference Unit, National Institute for Communicable Diseases, National Health Laboratory Service, Private Bag X4, Sandringham, Johannesburg 2131, South Africa; 3 Foray Consultants, P.O. Box 383, Hillcrest, 3650, KwaZulu-Natal, South Africa; 4 Maragra (Illovo) Sugar Estate, Manhica, Mozambique

## Abstract

**Background:**

The dual problems of rising insecticide resistance in the malaria vectors and increasing human malaria cases since 2001 in southern Mozambique are cause for serious concern. The selection of insecticides for use in indoor residual spraying (IRS) programmes is highly dependent on the extent to which local mosquitoes are susceptible to the approved classes of insecticides. The insecticide resistance status and role in malaria transmission of *Anopheles funestus *was evaluated at the Maragra Sugar Estate in southern Mozambique where an IRS vector control programme has been in operation for seven years using the carbamate insecticide bendiocarb.

**Results:**

No *Anopheles *species were captured inside the sugar estate control area. *Anopheles funestus *group captured outside of the estate represented 90% (n = 475) of the total collections. Of the specimens identified to species by PCR (n = 167), 95% were *An. funestus s.s. *One *An. rivulorum *was identified and seven specimens did not amplify. The *Anopheles gambiae *complex was less abundant (n = 53) and of those identified (n = 33) 76% were *An. arabiensis *and 24% *An. merus*. Insecticide susceptibility tests showed that wild-caught and F-1 family *An. funestus *were resistant to deltamethrin (32.5% mortality) and lambda-cyhalothrin (14.6% mortality), less so to bendiocarb (71.5% mortality) and fully susceptible to both malathion and DDT (100%). Bendiocarb and pyrethroid resistance was nullified using 4% piperonyl butoxide (Pbo), strongly suggesting that both are mediated by P450 monooxygenase detoxification. ELISA tests *of An. funestus *for *Plasmodium falciparum*, gave a sporozoite rate of 6.02% (n = 166). One unidentified member of the *An. gambiae *complex tested positive for *P. falciparum *sporozoites.

**Conclusion:**

*Anopheles funestus *was found to be the most abundant and principle vector of malaria in this area, with members of the *An. gambiae *complex being secondary vectors. Despite the continual use of bendiocarb within the estate for seven years and the level of *An. funestus *resistance to this insecticide, the IVC programme is still effective against this and other *Anopheles *in that no vectors were found inside the control area. However, the Mozambique National Malaria Control Programme ceased the use of DDT and bendiocarb in this area of its operations in 2009, and replaced these insecticides with a pyrethroid which will increase insecticide resistance selection pressure and impact on control programmes such as the Maragra IVC.

## Background

Malaria in the south of Mozambique is mesoendemic to hyperendemic and is a major medical and socio-economic burden to the country and the primary cause of clinic outpatient attendance [1]. It impacts particularly on the morbidity and mortality of children <5yr of age. Virtually all the population of Mozambique (99% of 20.8 million people) is at risk of malaria, with 3.4 million children <5 yrs being the most vulnerable [1]. Malaria is on the increase in Mozambique with 4 million cases in 2001 and 6 million cases in 2006. *Plasmodium falciparum *accounts for 90% of parasite infections and *P. malariae *and *P. ovale *for 9% and 1% respectively [1].

Malaria control using indoor residual spraying (IRS) started in Mozambique in 1946, using DDT and Benzene Hexachloride (BHC) and stopped in 1956, with good results in reducing parasite and spleen rates in children <5 yrs during that period. Control actions were initiated again in 1960 using DDT as part of a malaria eradication programme and continued through to 1971, when malaria control operations were limited to main towns due to civil war. By 1980 malaria control activities were confined to the Maputo area. A limited control action was again initiated in 1994 to evaluate lambda-cyhalothrin, deltamethrin, baythroid and cyfluthrin insecticides, all pyrethroids or derivatives of pyrethrum. Lambda-cyhalothrin was selected as the insecticide of choice at that time (reviewed by Casimiro [2]).

In October 1999 the first commercial integrated malaria vector control (IVC) programme in Mozambique was implemented at the Mozal aluminium smelter in the Beluluane district of Maputo, creating a buffer zone of 1.6 km (1mile) around the smelter as proposed by Charlwood et al. [3] initially using deltamethrin (RGK, unpublished data). In early 2000 an insecticide resistance study was carried out on *Anopheles funestus *at the Mozal site [4]. This was the first insecticide resistance study to be carried out in Mozambique and *An. funestus *proved to be resistant to both deltamethrin and lambda-cyhalothrin. Deltamethrin was replaced by the carbamate insecticide bendiocarb on the basis of vector susceptibility to this insecticide, it's acceptability for use in both western and traditional structures, and its non-repellency of mosquitoes. Shortly thereafter, following this work at Mozal, the Mozambique National Malaria Control Programme (MNMCP) under the auspices of the Lubombo Spatial Development Initiative (LSDI), also changed from pyrethroid insecticides to bendiocarb for IRS in southern Mozambique, and has continued to do so, with the addition of DDT for IRS in 2006 [5,6].

The IVC programme at Maragra Sugar Estate, 90 km north of the capital city Maputo, started in 2002. It encompasses the central estate residential and factory areas and the rural area within a radius of 1.6 km from the centre of the estate residential area, creating a barrier effect or *cordon sanitaire *around the target estate residential and mill areas. Due to the endophily and endophagy of *An. funestus*, the IVC management programme consists of a four month cycle of IRS with bendiocarb, to all homes, offices and factory, monthly IRS of the factory itself and fortnightly ultra-low-volume (ULV) spraying of the factory with the organophosphate insecticide dichlorvos to knockdown predominantly *Culex *spp. of mosquitoes. This escalation of IRS and ULV to the factory is due to the factory operating on a 24 hour basis during the harvesting and milling of the cane. Within the estate itself a programme of repairing and refurbishing drains and septic tanks and their covers is in place. Mosquito nets are provided to employees and their families on a voluntary and subsidized basis. The nets are not impregnated with a pyrethroid due to the high levels of pyrethroid resistance in the malaria vectors. Health education on personal protection measures, life cycle and habits of vector mosquitoes and the need for early diagnosis and treatment at the company and government clinic, is carried out on an informal and formal basis to expatriate and local people within the estate control area. Diagnosis by microscopy is carried out on all employees and expatriate family members presenting with symptoms, and treatment with an ACT is given on a positive slide diagnosis.

In the light of increased usage of pyrethroids by the MNMCP and the dropping of bendiocarb for IRS (http://www.rollbackmalaria.org/countryaction/mozambique_roadmap.html, 2009-2010), a survey was initiated to find out the level of resistance to pyrethroids and bendiocarb in the Maragra area.

## Materials and methods

### Study area

**Figure 1:**
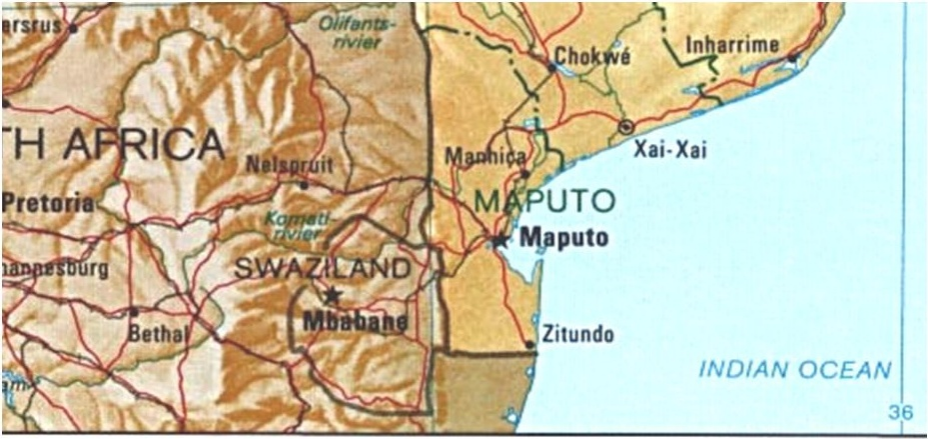
**Map of southern Mozambique showing Manhica north of the capital of Maputo**.

**Figure 2:**
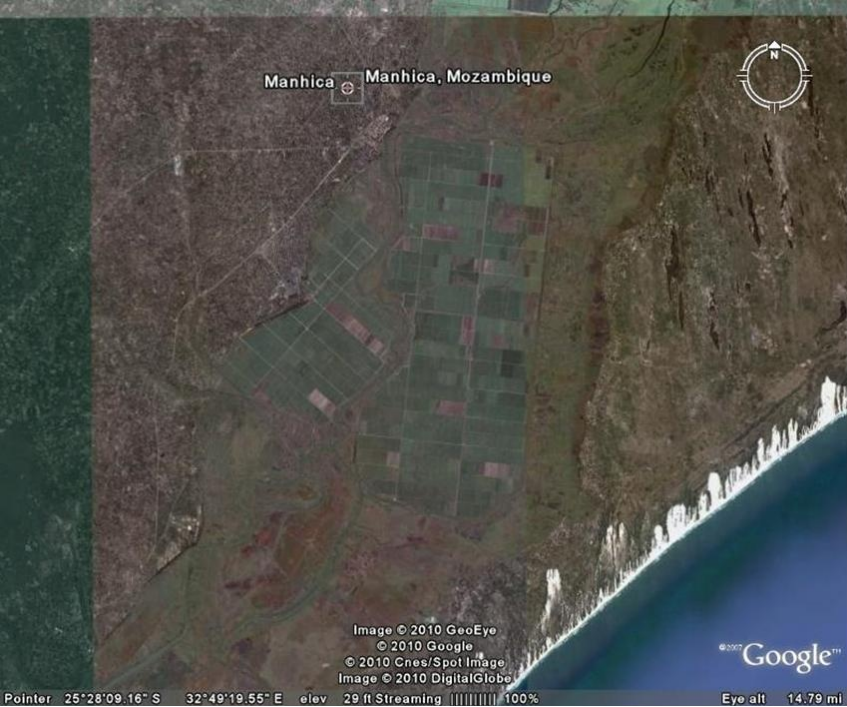
**Google Earth map showing the Maragra sugar estate south of Manhica in southern Mozambique**.

Maragra sugar estate is situated in the Maputo province of Mozambique (25°27'S, 32°46'E), 90 km north of Maputo city, 3 km south of Manhica town and approximately 10 km inland from the Indian Ocean (Figures 1, 2). It is surrounded on all but the east side by three rural villages forming one single unit with a population of ± 20,000. The number of Maragra estate employees fluctuates between 1,250 and 4,500 personnel per month through the year, with a significant increase in personnel from April through to November/December, when the harvesting and milling of the cane takes place. These personnel are drawn from the surrounding villages and other rural areas.

### Adult mosquito collections

The field site at Maragra was visited for two weeks per month from January to March 2009. Mosquitoes were sampled by indoor house searches, knock down collections, window exit traps, natural shelters and pit traps from areas inside and outside the sugar estate control operations. Live female *An. funestus *group and *An. gambiae *complex mosquitoes were placed in polystyrene cups and transported back to the Vector Control Reference Unit (VCRU) insectary at the National Institute for Communicable Diseases (NICD), Johannesburg. Subsequently, females were tubed individually for egg-laying and the egg batches reared under standard conditions to obtain F-1 adult progeny.

### Species identification

Captured mosquitoes were initially identified to group by morphology [7]. PCR methods for the *An. funestus *group [8] and *An. gambiae *complex [9] were used to identify the species in each group. Extraction of DNA [10] was done for the *An. funestus *group, and direct homogenization of a single leg for the *An. gambiae *complex.

### ELISA for P. falciparum parasites

Vector mosquitoes collected from an unsprayed area outside of the Maragra estate were assayed by enzyme-linked immunosorbent assay (ELISA) to investigate their *Plasmodium falciparum *sporozoite infection rate. This assay entailed homogenizing the mosquito head and thorax and measuring the *P. falciparum *circumsporozoite (CS) protein levels [11].

### Insecticide Susceptibility

Insecticide susceptibility bioassays were carried out on both wild caught and F1 generation mosquitoes to the four classes of approved public health insecticides, according to the standard WHO operating procedure [12]. Insecticides tested were: 4% DDT (organochlorine), 5% malathion (organophosphate), 0.1% bendiocarb and 0.1% propoxur (carbamates) and 0.05% deltamethrin and 0.05% lambda-cyhalothrin (pyrethroids). Bioassays were also conducted on bendiocarb and deltamethrin without and with the synergist piperonyl butoxide (4% Pbo).

Wild-caught females and 2-5 day old F-1 progeny were exposed to insecticide treated papers for one hour. Knockdown was recorded at the end of the exposure period and 24 hour mortality the following day. Between two and nine replicates per insecticide were performed dependent upon available mosquitoes, with an emphasis on bendiocarb as this is the insecticide in use on the estate at present. Controls were not exposed to any insecticide. The WHO insecticide treated papers were tested for efficacy by exposing a susceptible reference strain (*An. gambiae *SUA) to them in the same manner as for the exposed mosquitoes.

The Pbo experiments were conducted on 2-4 day old F-1 females. Mosquitoes were exposed to 4% Pbo-treated papers for one hour prior to exposure to deltamethrin and bendiocarb. Controls were exposed to Pbo only. Knockdown was recorded at the end of the one hour exposure and again at 24 hours for final mortality.

## Results

### Wild Mosquito Collections

A total of 528 wild caught vector mosquitoes were collected from the study site over the period January to April 2009 (Table 1). *Anopheles funestus *group was dominant in the collections (90%, n = 475) with the *An. gambiae *complex being present in very low numbers (10%, n = 53).

**Table 1:** *Anopheles funestus *and *An. gambiae s.l. *collection methods and species identifications

Method	*An. funestus*	*An. rivulorum*	*An. gambiae s.l.*	*An. arabiensis*	*An. merus*
Indoor Resting	439	1	38	25	8

Window Trap	9	0	5	-	-

Knockdown	26	0	10	-	-

Natural Shelters & Pit Traps	0	0	0	0	0

Total	474	1	53	25	8

Indoor house searches proved to be the most successful method with the majority of both *An. funestus *group and *An. gambiae *complex found resting indoors in unsprayed houses outside of the Maragra control area. Knockdown catches and window exit traps were not as effective, particularly for *An. funestus*. Indoor searches, knockdown catches and window traps in sprayed houses within the Maragra IVC resulted in no collections of vector mosquitoes resting indoors. Natural shelters and pit traps were entirely unsuccessful in the collection of vector mosquitoes, but highly successful for the collection of *Culex *spp. It was evident from this collection technique that both *An. funestus *group and *An. gambiae *complex do not utilise such refuges in this area.

### Species identification

A total of 175/475 (36%) of the *An. funestus *group and 52/53 (98%) of the *An. gambiae *complex were tested by PCR to establish species-specific identification. The majority of the *An. funestus *group samples were identified as *An. funestus s.s. *(95%; n = 167) and one specimen identified as *An. rivulorum*. Only 33/52 of the *An. gambiae *complex samples gave amplified products and of these 75.8% (n = 25) were identified as *An. arabiensis *and 24.2% (n = 8) as *An. merus*. The lack of PCR products from 19 samples could have been due to DNA degradation through poor storage in the field.

### ELISA assays for P. falciparum parasites

A total of 166 *An. funestus *samples were subjected to the ELISA test for *Plasmodium falciparum *circumsporozoite (CS) protein. Ten specimens were confirmed positive after retesting, giving a positivity rate of 6.02% for *An. funestus *in the areas outside of the Maragra IVC zone. The ELISA tests conducted on 52 *An. gambiae *complex samples gave one confirmed positive, i.e. 1.9%. Unfortunately, this specimen was not identified to species as it did not amplify on PCR.

### Insecticide resistance

Due to low numbers of the *An. gambiae *complex collected on a daily basis, insufficient females survived for egg-laying, and of those that did, oviposition was not successful. As a result no insecticide resistance assays could be carried out on this group of species.

A total of 952 *An. funestus *wild-caught and F-1 generation females (including controls) were used in the tests for susceptibility to the four classes of insecticides. The results are summarised in Table 2. Given that 99.4% of all PCR identified samples were *An. funestus s.s*., it is assumed that the results from this section pertain to this species. A total of 261 wild caught *An. funestus *were tested for susceptibility on the same day they were collected. The highest levels of resistance were found to lambda-cyhalothrin (14.6% mortality 24-hr post-exposure) and deltamethrin (32.5% mortality) (Table 2). According to WHO criteria, resistance to bendiocarb with 71.2% mortality in the wild caught population was confirmed. Using F-1 generation females, similar low mortalities were found for pyrethroids and bendiocarb (Table 2). The Chi-square values for comparisons between the wild samples and the F-1 generation 2-5 day old mosquitoes were deltamethrin 5.15 (p < 0.05), lambda-cyhalothin 3.94 (p < 0.05) and bendiocarb 0.11 (p > 0.05). This indicates that at least for the pyrethroids there is a significant difference in survival between wild-caught mosquitoes and the F-1 laboratory reared progeny, with wild-caught females surviving better on exposure to pyrethroids. Both DDT and malathion gave 100% mortality (Table 2).

**Table 2:** Insecticide susceptibility of *Anopheles funestus s.s. *from Maragra, Mozambique

Insecticide	Wild-caught		2-5 day old F-1 progeny	
	**No. Exposed**	**% Mortality**	**No. Exposed**	**% Mortality**

0.05% Deltamethrin	37	32.5	156	52.6

0.05% Lambdacyhalothrin	35	14.6	54	33.3

0.1% Bendiocarb	117	71.2	76	72.4

4% DDT	20	100	61	100

5% Malathion	52	100	52	100

Those F-1 families producing enough adults for subsequent analysis were assayed either individually or pooled against the monooxygenase inhibitor Pbo and the insecticides bendiocarb and deltamethrin (Table 3). In all samples 100% susceptibility to bendiocarb was achieved with the Pbo and 92.6% to deltamethrin. The deltamethrin results are in agreement with previous studies [4,13] while the bendiocarb results strongly suggest monooxygenase detoxification as well.

**Table 3:** Percent mortality of Piperonyl butoxide (Pbo) synergised and unsynergised F-1 samples of *Anopheles funestus *from Maragra, Mozambique.

	Pooled F-1	Family No. 19	Family No. 23	Family No. 32	Family No. 39	Family No. 42	Family No. 58
0.1% Bendiocarb	72.2 (18)	77.7 (9)	100 (6)	62.5 (8)	57 (7)	83 (12)	62.5 (16)

4% Pbo + 0.1% Bendiocarb	100 (19)	100 (10)	100 (9)	100 (8)	100 (7)	100 (12)	100 (16)

0.05% Deltamethrin	50 (42)	-	-	-	-	-	-

4% Pbo + 0.05% Deltamethrin	92.6 (27)	-	-	-	-	-	-

Controls 4% Pbo only	0 (184)						

Sample sizes in parenthesis.

## Discussion

While IRS within the Maragra IVC zone has been very effective in controlling both the *An. funestus *group and the *An. gambiae *complex, circulating adults of these species may be responsible for those malaria cases presenting within the IVC zone. Although the bendiocarb resistance in the wild populations of *An. funestus *around Maragra is cause for concern, spraying the 1.6 km buffer zone (as recommended by [3] and [14]) with this insecticide appears to be exerting an effective control influence as no vectors were found resting indoors or collected in houses from the IVC area. In contrast, all mosquitoes collected during this study were found outside the Maragra IVC area, with indoor resting *An. funestus *being the most common vector present.

The results of the insecticide bioassay trials reflect a similar pattern of resistance to pyrethroids and carbamates that has already been reported for southern African populations of *An. funestus *[5,15]. The results for the WHO susceptibility tests on wild female mosquitoes of unknown age when compared with 2-5 day old F-1 laboratory reared progeny, revealed that there is a statistically significant survival of wild (i.e. older) mosquitoes. This is in contrast to *Anopheles gambiae s.s. *where higher frequencies of resistance are expected in younger mosquitoes because of the fitness cost to older mosquitoes carrying the resistance genes [16].

Aranda et al. [17] surveyed the vector populations in the Manhica area from October 1997 to September 1998 and reported peak *P. falciparum *sporozoite rates of 5% in November and >2% in May. These rates are slightly lower than those reported in this study (6%), which was carried out during the mid to latter part of the rainy season (January to March). However, their main method of collection was different in that CDC light traps were used which may have attracted a different segment of the population to those collected passively resting inside houses in the present study. What is apparent, however, in comparing the two studies, is that the MNMCP operations in this area have had very little impact on the vector populations over the past 11 years.

Further investigations need to be done with regard to the breeding sites and role of malaria transmission of the *An. gambiae *complex in this area. A recent study carried out in Boane, outside the capital Maputo, showed that *An. merus *can play a significant role in malaria transmission in this region [18], this being the first record from southern Africa. Many searches for larval habitats have been carried out in the Maragra area in the past without success (RGK personal observations). Salt from the sea during the Cretaceous, Miocene and Pliocene times [19] may have leached to the surface due to the irrigation practices in these areas, creating suitable saltwater breeding sites for *An. merus*. However, *An. merus *has previously been found in the same freshwater bodies as other members of the *An. gambiae *complex, albeit far inland [20,21], so saltwater habitats are not necessarily a pre-requisite for this species.

Mayor et al. [22] reported that almost half of the human adult population from Manhica town were positive for *P. falciparum *during the dry season, and that rates may be even higher in the wet season. Mabunda et al. [23] reported human malaria parasite prevalence of 58.9% of which 52.4% were due to *P. falciparum*, the burden being in the northern areas and also the coastal areas, where Maragra is situated. The high sporozoite rates found in *An. funestus *in this study would support findings on high levels of parasitaemia in the human populations.

Despite the use of bendiocarb, lambda-cyhalothrin and DDT in the areas outside of the Maragra IVC, personal observations showed that not all the dwellings had been sprayed, and in many instances those that had been sprayed were not done so in accordance with WHO guidelines on IRS [24]. Insecticide deposits on the walls were erratic in concentration and distribution over substrates and dwellings (RGK, personal observations). Partial explanation for this may be due to the subsistence agricultural activities of the community. People are tending to their fields very early in the morning to escape the heat later in the day. When the spray teams arrive they often find locked homes, or the head of the household is not present and, due to local custom, entrance to the dwellings is denied by other members of the family. Supervision of the spray schedules and follow up on IRS coverage may also be lacking in some aspects. The integrity of the spray programme is then compromised to the detriment of the malaria control efforts [25,26].

An added problem is the leakage of insecticides from government storage facilities [27] and the use of these in domestic households or by subsistence farmers in agriculture. These issues, together with the uneven and erratic IRS coverage mentioned above, are almost certainly exerting strong selective pressure on the *An. funestus *populations. The vector control operations that are ongoing outside of the Maragra IVC area are obviously not having any positive impact on malaria transmission, given the 6% sporozoite rate in *An. funestus*. Continued monitoring and urgent improvement of this situation is critical to the success of both the Maragra IVC and the MNMCP as the arsenal of new, available and acceptable insecticides is extremely limited [28,29]. Moreover, the recent policy decision by Mozambique to use only lambda-cyhalothrin for IRS [27] should be reconsidered in the light of the results presented here and by earlier research [4-6].

## Conclusions

The success of the Maragra IVC programme is due in large part to a 'captured population' where spraying of all dwellings, offices and the mill is mandatory and adheres to a four monthly cycle of IRS. This includes the surrounding village areas falling within the 1.6 km buffer zone, compliance being ensured by the chief of the particular area. There are financial implications of this strategy to the sugar estate, but they are outweighed by the low levels of malaria disease burden and increased health of the community and workforce. In this instance, Maragra is implementing a leading and excellent corporate and social obligation and responsibility to its employees and the surrounding local community, through sustainable control of malaria and its vectors.

## Competing interests

The authors declare that they have no competing interests.

## Authors' contributions

RGK carried out the study, processed the specimens and drafted the manuscript. EN assisted with the field work and specimen collection and processing. RHH and MC designed the study, assisted with analysis of the data and helped draft the manuscript.
